# ChemDIS 2: an update of chemical-disease inference system

**DOI:** 10.1093/database/bay077

**Published:** 2018-07-23

**Authors:** Chun-Wei Tung, Shan-Shan Wang

**Affiliations:** 1 School of Pharmacy, Kaohsiung Medical University, 100 Shih-Chuan 1st Road, Kaohsiung, Taiwan; 2 PhD Program in Toxicology, Kaohsiung Medical University, 100 Shih-Chuan 1st Road, Kaohsiung, Taiwan; 3 Research Center for Environmental Medicine, Kaohsiung Medical University, 100 Shih-Chuan 1st Road, Kaohsiung, Taiwan; 4 Department of Medical Research, Kaohsiung Medical University Hospital, 100 Tzyou 1st Road, Kaohsiung, Taiwan; 5 National Institute of Environmental Health Sciences, National Health Research Institutes, 35 Keyan Road, Zhunan, Miaoli County, Taiwan

## Abstract

Computational inference of affected functions, pathways and diseases for chemicals could largely accelerate the evaluation of potential effects of chemical exposure on human beings. Previously, we have developed a ChemDIS system utilizing information of interacting targets for chemical-disease inference. With the target information, testable hypotheses can be generated for experimental validation. In this work, we present an update of ChemDIS 2 system featured with more updated datasets and several new functions, including (i) custom enrichment analysis function for single omics data; (ii) multi-omics analysis function for joint analysis of multi-omics data; (iii) mixture analysis function for the identification of interaction and overall effects; (iv) web application programming interface (API) for programmed access to ChemDIS 2. The updated ChemDIS 2 system capable of analyzing more than 430 000 chemicals is expected to be useful for both drug development and risk assessment of environmental chemicals.

Database URL: ChemDIS 2 is freely accessible via https://cwtung.kmu.edu.tw/chemdis

## Introduction

The identification of chemical-induced effects on human beings is vital for both the drug development and chemical risk assessment. The application of traditional experimental methods to fully characterization of chemical-induced health effects could take several years and require enormous resources for a single chemical. Due to the huge number of chemicals that may cause diseases, the use of high-throughput methods for chemical prioritization can greatly save resources and time. Various modern experimental approaches have been developed for this purpose. For example, large-scale omics projects such as DrugMatrix (1), CMAP (2, 3) and TG-GATES (4) provide useful tools for the investigation of chemical-induced effects on several selected cells, tissues and organs. Tox21 program starts from 2008 aiming to develop high-throughput screening assays to accelerate the study of chemical-induced effects (5). The biochemical- and cell-based assays could help the fast prioritization of chemicals for detailed toxicological evaluation and mechanism study. However, the abovementioned experimental methods are both time-consuming and expensive. Given the extremely large chemical space, the development of computational methods is desirable for the prioritization of chemical-induced effects and identification of potentially affected organs for further experimental investigation.

To address the need for fast analysis of chemical-induced effects, a computational system named ChemDIS for chemical-disease inference has been developed for analyzing >90 000 chemicals including many poorly characterized chemicals such as maleic acid and sibutramine (6, 7). The integration of multiple large-scale databases of chemical–protein interactions and functional annotations of genes and enrichment analysis methods enables the analysis of enriched functions, pathways and diseases for studying chemical-induced effects. Integrated ontology databases include Gene Ontology (GO) (8), Reactome (9), KEGG (10), Disease Ontology (DO) (11) and Disease Ontology Lite (DOLite) (12). As a successful application, the potential effects of maleic acid on neuronal functions have been demonstrated (13). As data is growing fast, it is desirable to extend the applicability of ChemDIS to more chemicals.

In this report, we present a new ChemDIS 2 system with four major improvements. First, the applicability has been extended to include >430 000 chemicals and >15 million chemical–protein interactions based on STITCH 5 (14). Second, both frontend and backend programs have been completely rewritten to give faster analysis performance. Third, a useful pathway database SMPDB (15) has been integrated into ChemDIS 2 providing more comprehensive analyses of enriched pathways. Fourth, a web application programming interface (API) has been developed for the programmatic access to functions of ChemDIS 2.

## Integrated resources and implementation of ChemDIS 2

The previous version of ChemDIS was based on the integration of R packages and related SQLite databases. While a Rserv server was implemented in ChemDIS to accelerate computational performance, it is no longer suitable for dealing with the largely increased dataset in ChemDIS 2. In this work, we have implemented all programs using GO language and a modern NoSQL database of MongoDB supporting the fast calculation, high-performance access to data and web APIs. Database and programs were deployed in an Ubuntu server.

To enable the inference of chemical-induced effects, several databases were downloaded and integrated into a MongoDB database including STITCH 5 (14), Reactome (9), SMPDB (15), miRTarBase (16), Ensemble (17), DOSE (18), DO.db (19), KEGG.db (20) and org.Hs.eg.db (21). The versions of databases will be updated periodically and shown in the manual of ChemDIS 2 (https://chun-weitung.gitbooks.io/chemdis/content/data-sources.html). Currently, >430 000 chemicals with >15 million chemical–protein interactions can be analyzed using ChemDIS 2 that is >3 times larger than the original ChemDIS system.

**Figure 1.**
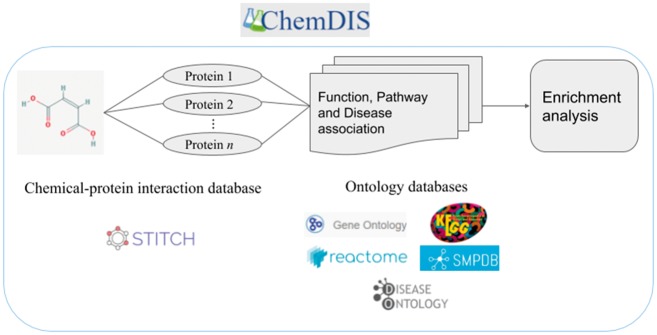
The flowchart of ChemDIS system and associated data sources.

The flowchart of ChemDIS 2 for single chemical analysis is shown in Figure 1. Given a test chemical, its interacting proteins will be extracted from STITCH data. Subsequently, Ensemble was utilized for mapping the protein identifiers to Entrez gene IDs. To extract functional annotations of the gene IDs, the associated ontology terms were extracted from databases of org.Hs.eg.db, GO, Reactome, KEGG.db, SMPDB and DOSE (DO and DOLite). Finally, enrichment analyses will be conducted based on the chemical-protein-ontology associations. The supported ontology databases include GO, KEGG, Reactome, SMPDB, DO and DOLite.

## User interface

**Figure 2.**
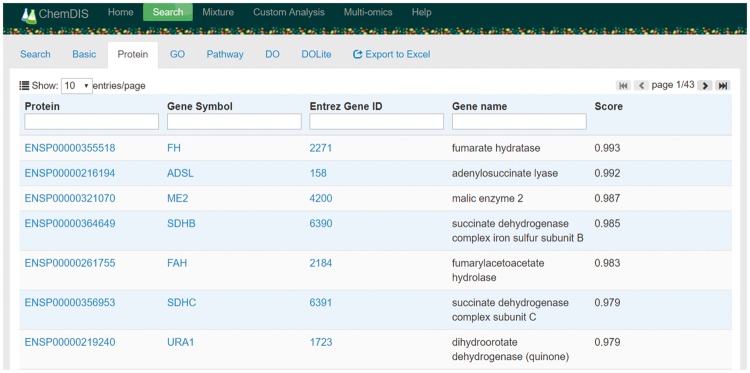
A screenshot of ChemDIS system for the analysis of interacting proteins for maleic acid. All columns are sortable by clicking the column name. Columns with a search box are searchable. Hyperlinks to external databases of Ensemble and GenBank enable the exploration of further information. The button of ‘Export to Excel’ will appear once all analyses have been done.

The user interface has been redesigned based on AngularJS providing better user experiences in ChemDIS 2 as shown in Figure 2. Chemical name and Chemical Abstracts Service (CAS) number are acceptable input for conducting an analysis in ChemDIS 2 with the help of a autocomplete function. Once the analysis is done, corresponding tabs will become clickable for accessing results of interacting proteins and enriched terms of GO, pathway and DO. Please note that the analysis for a given compound is conducted in a real-time manner that will be stored in a temporary database for quick access. The temporary database will be erased on the update of any data sources to keep all calculations up-to-date. Hyperlinks to external databases such as Ensemble, GenBank (22), PubChem (23), GO and DO were included in the result table. Please note that the DOLite website is no longer functional and the DOLite analysis will be removed in the next major release. All the results can be exported and downloaded as an EXCEL file consisting of tabs for basic information, interacting proteins, and enriched functions, pathways and diseases. Individual EXCEL files, each corresponding to one of the tabs, are also included.

In addition to the major update to the search function for chemical-protein-disease association inference, ChemDIS 2 offers three useful tools to help the analysis of chemical-induced effects including functions for custom, multi-omics and mixture analyses. Instead of relying on chemical–protein interaction data from STITCH database, the custom analysis function accepts user inputs of a custom set of differentially expressed genes, proteins, miRNAs or metabolites that could be derived from various omics experiments, and enrichment analysis will be conducted. Significantly affected functions, pathways and diseases will be identified for the user-supplied set of gene, miRNA or protein identifiers. For example, our previous work conducted gene expression profiling of maleic acid-treated neuronal cells and identified differentially expressed genes whose associated functions were analyzed based on the custom analysis function (13). The server currently accepts identifiers of Entrez gene ID, Ensembl protein ID, Ensembl gene ID, Ensembl transcript ID, Pfam ID, UniProt accession number and RefSeq accession number. For inputs of chemical identifiers including chemical names and CAS numbers, only enriched pathways will be identified since the associations between chemicals and functions/diseases have not yet been defined. For inputs of miRNAs, their experimentally validated targets will be mapped based on miRTarBase and will be analyzed for enriched functions, pathways and diseases. The results of custom analysis will be stored in the temporary database for one week and can be retrieved by a unique ID.

The emerging multi-omics approaches are promising for studying diseases by integrating individual results from single omics experiments (24). To support modern multi-omics projects that generate a few kinds of omics data, we have developed a multi-omics tool for joint analysis of multi-omics data. The multi-omics function is basically an extension of custom analysis that accepts up to four sets of user-supplied genes, proteins, miRNAs or metabolites. The resulting individual analysis results will then be jointly analyzed to identify consensus effects supported by multiple evidence. A joint *p*-value $p_{j}=\prod_{i=1}^{n}p_{i}$ will be calculated representing the overall significance for each term, where *p_i_* represents the adjusted *p*-value for dataset *i*. The joint *p*-value has been shown to be effective for the identification of enriched terms supported by multiple datasets (25). The results will be kept in the temporary database for one week and can be retrieved by a unique ID. We are working on the next update to increase the number of sets for multi-omics analysis that will be expected to be available in the next few months.

For the analysis of effects by exposure to multiple chemicals, the mixture function was designed for the analysis of shared interacting proteins, potential interacting effects and overall effects(26). The analysis results will be shown as Venn diagram charts representing the common interacting effects and unique effects for each chemical. Similarly, a joint *p*-value *p_j_*will be calculated for prioritizing the interacting effects.

### Web API

**Figure 3.**
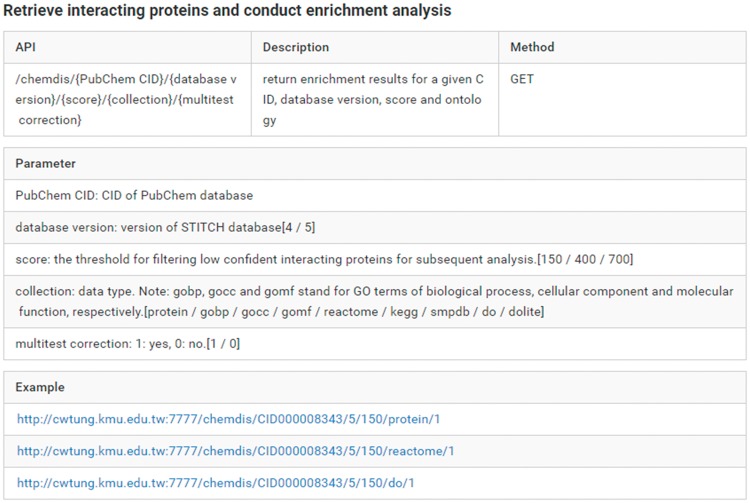
The manual of web API for accessing ChemDIS functions. The manual gives detailed information of the API, description, method, parameters and examples. The analysis results will be returned as a JSON object.

While ChemDIS 2 is equipped with an easy user interface, there is an increasing need for programmatic access to core functions of ChemDIS for streamlining analysis workflow and implementation of new servers incorporating the analysis functions. To improve the interoperability of ChemDIS 2, a new web-based application programming interface (API) was implemented using Go language for accessing core functions such as the identification of interacting proteins and chemical-diseases associations. The web API is freely accessible via simple HTTPS calls. For example, given the PubChem CID of interest along with selected database version and a confident threshold for retrieving interacting proteins, the server will return a JSON (JavaScript Object Notation) object consisting of all analysis results. As an increasing number of functions are being developed, please refer to the online manual (https://chun-weitung.gitbooks.io/chemdis/content/web-api.html) for detailed information and examples of currently available web API. An example of web API is shown in Figure 3. A JSON object will be returned for subsequent analysis.

### Conclusion and future development

We present an update of ChemDIS system for the identification of interacting targets and inference of functions, pathways and diseases affected by chemicals. Databases have been updated to enable the analysis of >430 000 chemicals. The frontend and backend programs have been reimplemented to improve the efficiency of data analysis for the fast-growing data. New functions including the analysis of interaction and overall effects of mixtures and enrichment analysis of single omics and multi-omics results are expected to be useful for *in silico* analysis of chemical-induced effects based on our databases and experimental data. Web APIs were developed for programmed access to the major analysis functions of ChemDIS 2. Future works include the development and integration of target prediction methods to improve the ChemDIS analysis results based on a more comprehensive set of interacting targets and enable the analysis of chemicals without known interacting targets.

## Funding

This work was supported by Ministry of Science and Technology of Taiwan (MOST104-2221-E-037-001-MY3); National Health Research Institutes (NHRI-107A1-EMCO-0318184); and Research Center for Environmental Medicine in Kaohsiung Medical University from The Featured Areas Research Center Program within the framework of the Higher Education Sprout Project by the Ministry of Education (MOE) in Taiwan. Open access publication was supported by Ministry of Science and Technology of Taiwan (MOST104-2221-E-037-001-MY3). The funding agencies play no role in the study design, data analysis and manuscript preparation.

*Conflict of interest.* None declared.
